# Taurine in Septic Critically Ill Patients: Plasma versus Blood

**DOI:** 10.34172/apb.2023.015

**Published:** 2021-10-09

**Authors:** Ata Mahmoodpoor, Afsaneh Farjami, Niloufar Farzan, Hamed Hamishehkar, Parina Asgharian, Sarvin Sanaie, Kamran Shadvar, Farnaz Naeimzadeh, Hadi Hamishehkar

**Affiliations:** ^1^Department of Anesthesiology, Tabriz University of Medical Sciences, Tabriz, Iran.; ^2^Food and Drug Safety Research Center, Tabriz University of Medical Sciences, Tabriz, Iran.; ^3^Pharmaceutical Analysis Research Center, Tabriz University of Medical Sciences, Tabriz, Iran.; ^4^Iranian Evidence Based Medicine Center of Excellence, Tabriz University of Medical Sciences, Tabriz, Iran.; ^5^Drug Applied Research Center, Tabriz University of Medical Sciences, Tabriz, Iran.; ^6^Neurosciences Research Center, Aging Research Institute, Tabriz University of Medical Sciences, Tabriz, Iran.; ^7^Student Research Committee, Tabriz University of Medical Sciences, Tabriz, Iran.; ^8^Clinical Research Development Unit of Imam Reza Hospital, Tabriz University of Medical Sciences, Tabriz, Iran.

**Keywords:** Critically ill, Sepsis, Taurine

## Abstract

***Purpose:*** Sepsis and systemic inflammatory response syndrome (SIRS) encompass various problems throughout the body, and two of its major problems are the creation of oxidative substances in the body and decrease of the body’s antioxidant capacity to deal with the stress and organ damage. Optimal enteral nutrition fortified with antioxidant or immunomodulator amino acid is a hot topic concerning sepsis in the critical care setting. Taurine plays a protective role as an antioxidant in cells that is likely to have a protective role in inflammation and cytotoxicity.

***Methods:*** In the present study, 20 septic patients and 20 healthy volunteers were enrolled. The blood and plasma taurine levels of the patients on days 1, 3 and 7 were measured. Blood and plasma taurine level and the correlation between them, organ failure, and severity of the disease were assessed.

***Results:*** Taurine concentrations in the plasma of the septic patients were significantly lower than control group, and the whole blood concentrations were significantly higher than those of the control group (*P*<0.001). There was not a significant correlation between the blood and plasma taurine levels in control and septic patients. In addition, there was not any correlation between the severity of the disease, organ failure, mortality, and plasma as well as the blood concentration of taurine.

***Conclusion:*** In septic patients, taurine concentration in plasma and blood are low and high, respectively. These concentrations are not linked to each other and not associated with the patients’ outcome, and the disease severity, and organ failure.

## Introduction

 Oxidative injuries and inflammation are two main issues in sepsis. In addition to prescribing antibiotics for the treatment of sepsis, the use of supplements to control the mortality of sepsis has been considered and studied as well.^1-4^ Taurine as an imperative supplement, is a sulfonated amino acid derived from methionine and cysteine metabolism, and it has been suggested to have different functions such as membrane stabilization, osmoregulation, bile salt formation, and modulation of intracellular calcium homeostasis^5,6^ and is present in lymphocytes^7,8^ have abandon reserve of taurine which takes part in modulation of immune cell functions, protect against oxidative stress^9,10^ and regulate proinflammatory cytokines.^11^ Taurine therapy may also have potential advantages in reducing the destruction of neutrophils and tissue damage due to sepsis, which is unrelated to antioxidant effects.^12^

 Taurine is not incorporated into proteins, which may dissociate taurine levels from changes in protein synthetic and catabolic rates, and from the consequent changes in the plasma amino acid pool.^13^ Changes in osmotic imbalance, cell proliferation, and hepatic encephalopathy may cause changes in taurine concentration.^14^

 Previous studies have reported conflicting and contradictory results from changes in the concentration of taurine in blood and plasma in sepsis.^15-20^ However, taurine was suggested to be a utile and effective supplement, which may be indicated in sepsis treatment.^20^

 The primary goal of this research was to evaluate the change and correlation in the plasma and whole blood concentration of taurine over time in severe septic patients, and as the second goal, to study the relation between taurine concentration, clinical data, and outcome.

## Methods

###  Patients and study protocol 

 In the present prospective case-control study, 20 consecutive critically septic patients over 18 years within 24 hours after intensive care unit (ICU) admission and under mechanical ventilation were included. Sepsis is a syndrome caused by a disruption of the host’s immune response to infection includes abnormal physiological and biological abnormalities.^21^ All of the included patients were admitted to the 12-bed close format general ICU of a university-affiliated hospital from April 2015 to July 2016. The blood samples were taken from 20 healthy volunteers as the control group.

 The criteria for enrollment in the septic group was the presence of at least one positive culture (blood, urine, tracheal aspirates, wound, and CSF fluid) in addition to having systemic inflammatory response syndrome (SIRS) symptoms. The negative culture even with a high probability of sepsis was excluded from the study. Patient enrollment was based on the presence of at least two of the following SIRS criteria: body temperature ( > 38°C or < 36°C), tachycardia > 90 beats/min, respiratory rate > 20 breaths/min or Paco2 < 32 torr, white blood cell count > 12 000 cells/µL or < 4000 cells/µL, or > 10% immature (band) forms. Exclusion criteria were pregnancy, hematologic malignancy, agranulocytosis, intolerance to enteral nutrition, and cirrhosis. In the early hospitalization, demographic characteristics (weight, age, sex, body mass index) were recorded for all the patients, and central venous lines and Foley catheters were established, and all the patients were monitored for ECG and pulse oximetry. The illness severity and organ dysfunction were calculated by the Acute Physiology and Chronic Health (APACHE II) score^22^ and Sequential Organ Failure Assessment (SOFA) score,^23^ respectively. All the patients were feed via a Nasogastric tube. Pre-formulated standard enteral nutrition (Ensure^®^, Abbot, United States) was started at 20 ml/h, and the dose was increased by 20 ml/h if the gastric retention volume remained < 150 ml/h. In the case of intolerance, intravenous (IV) metoclopramide was initiated. Patients with a lack of tolerance to enteral nutrition for more than 24 hours were excluded from the study.

 Total energy expenditure was calculated using the Harris–Benedict equation. No vitamins, supplements and trace elements were added to the patients’ daily nutrition support.

 To measure taurine concentrations in blood and plasma, 5 mL of blood from the patients was taken on days 1 (during 24 hours), 3, and 7 after diagnosis of sepsis. Samples were divided into two parts, one part as whole blood restored and another part was centrifuged (PIT 320, Pole Ideal Tajhiz Co^®^) and plasma was separated. All the samples were frozen at -70 °C (NF305 AEG, Himalia^®^).

###  Taurine assay method

 Kamp and colleagues,^24^ modified the method by HPLC with a fluorescence detector was used to measure taurine levels. To calibrate the standard curve, 10 mL solution of taurine (10 mg) in 50% methanol was prepared. Five different taurine concentrations (10, 20, 40, 60, 80 and 100 μg/mL in water) were prepared and used as calibrators. When the samples were defrosted, then 100 μL the standard solution and 100 μL plasma were mixed for 30 seconds on the vortex (LS-100, Labtron^®^) then remained about 20 minutes at the lab temperature. Subsequently, it was diluted (1:10 v/v) in methanol then centrifuged for 5 minutes at a speed of 12 000 rpm and the supernatant was separated. Finally, 250 µL methanol solution with 200 µL of borate buffer (618 mg boric acid in 100 mL water, pH = 10 with NaOH), 250 µL methanol, 250 μL o-phthalaldehyde (OPA) (20 mg/mL in methanol) and 50 µL 3-mercapto-propionic acid (MPA) (in fume hood) were prepared and then kept in dark for 2 minutes. Correspondingly, prepared samples were subjected to HPLC apparatus (column: KNAUER C18 4 µm 15 cm, mobile phase: 85% disodium hydrogen phosphate 0.0125M and 15%).

 To calibrate blood samples, 100 µL blood, 100 µL solution with standard concentrations and 800 acetone were mixed and centrifuged for 5 minutes at 12 000 rpm. Then, 125 µL over solution, 125 µL methanol, 120 µL, 50 µL OPA borate buffer and 25 µL MPA were mixed for 30 seconds on the vortex and after two minutes, 20 µL was injected. This method was used for all concentrations.

###  Statistical analysis

 The results of the study conducted by Chiarla et al^13^ were used for sample size estimation. Assuming α = 0.05 and power = 0.9, the sample size of less than 10 was calculated using the software Power & Sample Size (PS), Version 3/0. Supposing 30% changes in laboratory parameters, to increase the study reliability, 20 septic patients and 20 healthy subjects were enrolled. We verified the existence of normality in the quantitative variables using the Kolmogorov-Smirnov test. Independent *t* test and analysis of variance (ANOVA), as well as chi-square test were used to compare quantitative and qualitative variables, respectively. The Pearson and Spearman correlations were used to evaluate the correlation between parametric and nonparametric data. Repeated measures analysis of variance was used to detect significant changes in the blood and plasma taurine concentration for patients during sequential measured times and also between dead and alive patients. A *P* value of less than 0.05 was considered statistically significant.

## Result and Discussion

 In this study, 41 patients were enrolled. Table 1 presents demographic data. Owing to death and transference to other wards, the number of patients on the third and seventh days dropped to 17 and 10 cases, respectively.

**Table 1:** Demographic, microbiological, and baseline data at the start of study

**Characteristics**	**Mean (±SD)**	***P*** ** value**
Age	-	0.054
Patients	60.7 ± 19.4	
Controls	50.9 ± 9.7	
Gender (male/female)	-	
Patients	10:10	0.65
Controls	11:9	
Weight	-	
Patients	78.6 ± 11.7	0.06
Controls	85.6 ± 11.4	
BMI	-	
Patients	25.2 ± 2.7	0.16
Controls	26.3 ± 2.5	
The reason for hospitalization	-	
Trauma	6	
Pulmonary Emboli	4	
Cerebrovascular disease	4	
Sepsis	3	
Post CPR	3	
Type of microbiology in septic patients	-	
Gram +	-	
*Staphylococcus aureus*	4	
*Staphylococcus epidermidis*	1	
Gram -	-	
*Escherichia coli*	3	
*Pseudomonas aeruginosa*	2	
*Acinetobacter baumannii*	5	
*Klebsiella pneumonia*	3	
Fungi	-	
*Candida albicans*	2	
APACHE II (admission)	20.7 ± 7.2	
SOFA (admission)	9.3 ± 2.5	

Data is presented as mean ± SD or number. APACHE II, Acute Physiology and Chronic Health Evaluation; BMI, body mass index; CPR, cardiopulmonary resuscitation; SOFA, Sequential Organ Failure Assessment.

**Figure 1:**
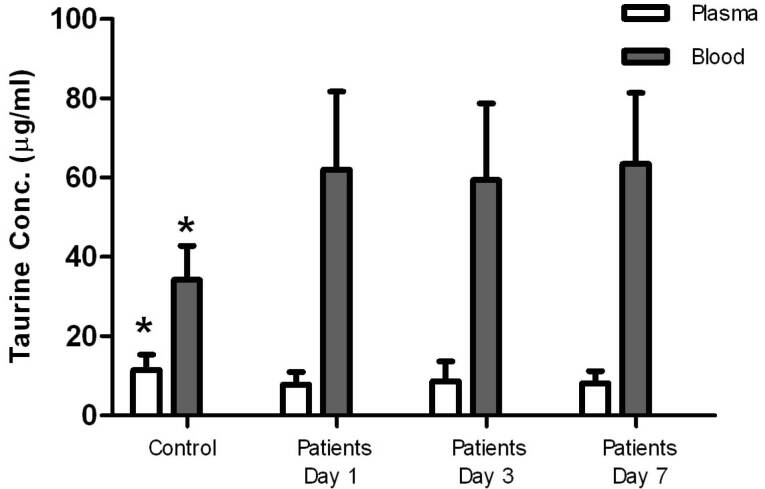
Taurine concentrations in plasma versus blood in the control group and the patients. This figure shows that taurine concentrations in plasma in the control group are significantly higher than those in the patients, but blood levels of taurine in the control group are significantly lower than those in the patients. In the patients’ taurine levels in blood and plasma on days 1, 3, 7, were not significantly different. *P*< 0.05 was compared with the measured taurine levels in the patients

**Figure 2:**
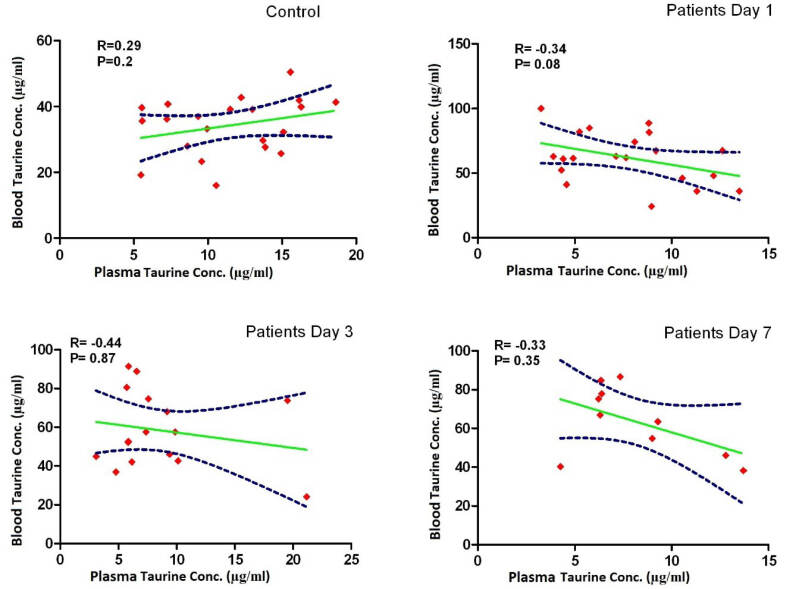
Correlation between taurine concentrations in plasma and blood in the control group and the patients on days 1, 3, 7. This figure shows that there is no significant correlation between the blood and plasma concentrations in the control group and the patients

**Figure 3:**
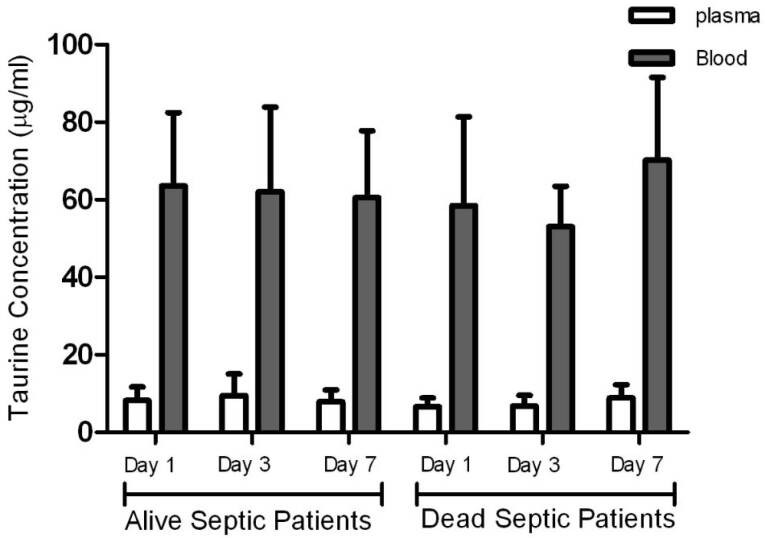
Taurine concentrations in plasma and blood in alive septic patients versus dead septic patients. This figure shows that there is no significant correlation between blood and plasma concentrations of the dead and alive patients

 Taurine concentrations in plasma in the control group were significantly higher than plasma concentrations measured in the patients at all times, but blood levels of taurine in the control group were significantly lower than those measured in the patients. Taurine levels in blood and plasma measured in the patients on days 1, 3, and 7 were not significantly different (Figure 1, Table 1), and there was no significant relationship between the blood and plasma concentrations in the control group and the patients (Figure 2, Table 2). In addition, repeated measurements analysis for plasma and blood taurine levels during the study days indicated no significant changes within patients (*P* = 0.48 and *P* = 0.97) (Figure 2, Table 2). Table 3 and Figure 3 present the plasma and blood concentration of taurine in dead and alive septic patients. There is no significant correlation between blood and plasma concentrations in dead and alive patients. Furthermore, repeated measurements analysis for plasma and blood taurine levels during the study days between dead and alive patients showed no significant change (*P* = 0.61 and *P* = 0.79) (Figure 3, Table 3)

**Table 2:** The taurine concentration in blood and plasma in patients and controls

	**Taurine concentration**	**N**	**Mean±SD ** **(µg/mL)**	***P*** value*
Patients	Plasma1	20	7.76 ± 3.15	0.002
	Plasma 3	16	8.62 ± 4.97	0.04
	Plasma 7	10	8.16 ± 3.04	0.03
	Blood 1	20	62.00 ± 19.73	0.000
	Blood 3	17	59.41 ± 19.31	0.000
	Blood 7	10	63.43 ± 17.93	0.000
Control	Plasma	20	11.43 ± 3.95	
	Blood	20	34.20 ± 8.54	

* Results based on *t *test comparing mean plasma and blood taurine concentration of patients with plasma and blood taurine concentration in control group.

**Table 3:** Taurine concentrations in blood and plasma in survived and died septic patients

	**Plasma 1 **	**Plasma 3 **	**Plasma 7 **	**Blood 1 **	**Blood 3 **	**Blood 7 **
Alive	8.24 ± 3.44	9.44 ± 5.63	7.88 ± 3.10	63.53 ± 18.93	62.07 ± 21.84	60.53 ± 17.26
Dead	6.63 ± 2.21	6.81 ± 2.75	8.84 ± 3.45	58.43 ± 22.94	53.02 ± 10.39	70.21 ± 21.32

 There was no correlation between sex, APACHE II score, SOFA score, vasopressor use and PaO2/FiO2 (as a respiratory index) with plasma and blood concentration of taurine. There was an opposite non-significant correlation between age and taurine plasma as well as blood concentration (Table 4).

**Table 4:** Correlation of age and the blood and plasma taurine concentrations in septic patients and control

**Age**		**Plasma 1**	**Plasma 3**	**Plasma 7**	**Blood 1**	**Blood 3**	**Blood7**
Patients	Correlation	-0.066	-0.012	-0.042	-0.231	-0.363	-0.382
	Sig. (2-tailed)	0.782	0.966	0.907	0.327	0.152	0.276
	N	20	17	10	20	17	10
Control	Correlation	0.396			0.205		
	Sig. (2-tailed)	0.075			0.372		
	N	20			20		

 There was no correlation between mean arterial pressure and blood and plasma concentrations in septic patients with taurine, but the correlation between taurine concentrations in plasma on days 3 and 7 and lactate and taurine concentrations in blood on day 7 in septic patients was statistically significant (Table 5).

**Table 5:** Correlation of age and the blood and plasma taurine concentrations in septic patients

		**Plasma 1**	**Blood 1**	**Plasma3**	**Blood 3**	**Plasma 7**	**Blood 7**
Lactate 1	Correlation	0.001	0.200				
	Sig. (2-tailed)	0.997	0.412				
Lactate3	Correlation			-0.741	0.115		
	Sig. (2-tailed)			0.001	0.661		
Lactate 7	Correlation					-0.618	0.673
	Sig. (2-tailed)					0.057	0.033
SOFA 1	Correlation	0.027	-0.077				
	Sig. (2-tailed)	0.909	0.48				
SOFA 3	Correlation			-0.289	-0.075		
	Sig. (2-tailed)			0.277	0.774		
SOFA 7	Correlation					-0.217	-0.292
	Sig. (2-tailed)					0.546	0.413
APACHE	Correlation	0.008	-0.172				
	Sig. (2-tailed)	0.975	0.467				
P/F 1	Correlation	0.027	-0.077				
	Sig. (2-tailed)	0.909	0.48				
P/F 3	Correlation			-0.289	-0.075		
	Sig. (2-tailed)			0.277	0.774		
P/F7	Correlation					-0.217	-0.292
	Sig. (2-tailed)					0.546	0.413
MAP 1	Correlation	-0.361	0.383				
	Sig. (2-tailed)	0.117	0.095				
MAP 3	Correlation			-0.226	0.118		
	Sig. (2-tailed)			0.401	0.651		
MAP7	Correlation					0.002	-0.513
	Sig. (2-tailed)					-.996	0.129
Vasopressor	Correlation	0.019	0.170	0.000	-0.211	0.342	-0.342
	Sig. (2-tailed)	0.94	0.473	1	0.417	0.334	0.334

APACHE II, Acute Physiology and Chronic Health Evaluation; MAP, mean arterial pressure; P/F, PaO2/FiO2 (as a respiratory index); SOFA, Sequential Organ Failure Assessment.

 Sepsis causes progressive damage in various organs. Septic shock progress with a significant reduction in blood pressure may lead to death.^25^ Although overall mortality of sepsis has declined over the last decade, it continues to be a disease associated with high mortality.^26^ It seems that antibiotics therapy is not an effective treatment to increase the chance of survival in septic patients.^25^ Taurine deficiency in the plasma of septic patients makes taurine as a complementary effective treatment.^13,20^

 Taurine is a free amino acid including two special features compared to other amino acids: i) No participation into proteins and peptides. ii) Unique intracellular transport system.^19^ It seems that intracellular and plasma concentrations of taurine in septic patients represent different variations through the disease process. Therefore, it is necessary to consider both intracellular and extracellular concentrations. Additional taurine in a normal diet excretes in the urine. In the case of limited access to taurine, kidneys increase reabsorption and decrease the excretion of taurine, leading to stable taurine level in the body.^27^

 The present study results indicated that the taurine concentration in the plasma of septic patients during 1, 3 and 7 days after entering the study was significantly lower than that of the control group. Furthermore, the blood taurine concentration was higher than that of the control group in all of samplings days.

 Paauw et al studied nine traumatic critical patients and showed that taurine concentration in the plasma of the patients was significantly reduced to 60% of that of the control group.^28^ This result demonstrated the necessity of taurine administration after injury.

 Engel et al tested 32 septic and traumatic patients and reported that taurine concentration in the plasma trauma was significantly reduced after trauma,^19^ supporting our experimental data. Although, Engel et al observed that taurine concentration was reduced in neutrophil cells, our results indicated that the taurine concentration in the patients’ blood and blood cells were higher than that of the control group. Neutrophils is the main component of white blood cells in a state of sepsis; therefore, the concentration of taurine in neutrophils is high. In their study, the reduced level of taurine in plasma and blood cells was maintained, and no significant change was observed compared to patients with trauma. Like ours, they did not find any correlation between plasma and intracellular concentrations.^19^ Vinton et al^29^ showed that the taurine level in plasma, platelets and white blood cells in critically ill patients admitted to the ICU was lower than that in healthy subjects, while the amount of taurine in granulocytes was stable, and no significant change was observed compared to the control group. Vente et al measured taurine level in plasma and other amino acids of 65 patients of whom 27 and 38 patients were septic and had SIRS, respectively.^17^ The results showed that the plasma concentration of amino acids, including taurine in septic and SIRS patients was significantly lower than that of the control group. The taurine level reduction in the plasma of septic and stressed patients had no significant difference. However, taurine level in severe sepsis was lower than the mild to moderate level. In the present study, taurine level in the blood and plasma of 6 died patients compared to 14 survived patients was not significantly different. It may be concluded that taurine level in blood or plasma is an inappropriate indicator to predict mortality or survival of septic patients. Taurine concentration in plasma is not higher than other amino acids concentration, but taurine intracellular level is often up to 10 times higher than other amino acids except glutamate.^30^ Taurine is approximately 76% and 50% of free amino acids into granulocytes and lymphocytes, respectively. Furthermore, a beta-amino acids transmitter system in lymphocytes maintains the high endogenous taurine level of plasma. Taurine therapy includes potential advantages in reduction of the neutrophils and tissue damage resulted from sepsis. These phenomena can be explained by antioxidant and membrane stabilizer effects of taurine. The important role of taurine in the immune system and anti-inflammatory effects with high doses in neutrophils and lymphocytes have been proposed.^31^ The activity of white blood cells and their need to promote the capacity of antioxidants and phagocytosis in sepsis justify the accumulation of plasma taurine in blood cells. This accumulation is probably due to its influx from plasma to white blood cells. Therefore, we cannot definitely claim that the lack of taurine in plasma is a logical reason for administration of taurine supplementation in septic patients since the taurine concentration in blood was higher than that of the control group. Furthermore, the lack of correlation between taurine concentration in plasma and blood supports this idea.

 In line with other studies, we found no correlation between SOFA score of septic patients and taurine level in plasma and neutrophils. Consequently, taurine levels are not associated with disease severity (APACHE II score). The results are inconsistent with the literature.^17,19,28^

 In the present study, no significant correlation between age, sex, and taurine concentration in blood and plasma were found. In agreement with the previous study,^18^ we found no correlation between arterial blood pressure and taurine level in blood and plasma. Furthermore, no correlation was found between taurine levels in plasma and blood and vasopressor as well as inotrope administration in septic patients and hemodynamic abnormalities. We evaluated respiratory insufficiency using the Po2/Fio2 index. Taurine levels in blood and plasma did not show any significant correlation with this index.

 The results showed a direct and significant correlation between taurine level in plasma and lactate level in blood on the third day. Furthermore, taurine level in blood and plasma was indirectly and significantly correlated with lactate level in blood on the seventh day. Chiarla et al^18^ demonstrated a significant correlation between taurine level in plasma and lactate level in blood. Lactate level in blood is a tissue hypoperfusion marker indicating sepsis severity. This level negatively and positively correlates with taurine levels in plasma and blood, respectively.

## Conclusion

 In septic patients, taurine concentration in plasma and blood are lower and higher, respectively comparing with healthy control population. These concentrations are not correlated to each other and also not correlated with the patients’ outcome, the disease severity and organ failure.

## Acknowledgments

 We thank Clinical Research Development Unit, Imam Reza General Hospital for their technical acceptance in manuscript preparation.

## Author Contributions

**Conceptualization: **Ata Mahmodpoor, Hadi Hamishehkar.

**Data curation:** Niloufar Farzan, Farnaz Naeimzadeh, Parina Asgharian.

**Data collection, patient recruitment:** Kamran shadvar, Ata Mahmoodpoor.

**samples Analysis**: Afsaneh Farjami, Hamed Hamishehkar.

**Investigation: ** Sarvin Sanaie, Parina asgharian.

**Methodology:** Ata Mahmodpoor, Hadi Hamishehkar, Sarvin Sanaie.

**Project administration:** Hadi Hamishehkar.

**Writing – original draft:** Niloufar Farzan, Parina Asgharian.

**Writing – review & editing:.** Farnaz Naeimzadeh.

## Ethical Issues

 This study is based on the principles outlined in the Declaration of Helsinki, and its ethical code was obtained from the Medical Ethics Committee. The written consent form was obtained from all the patients’ legal representatives.

## Conflict of Interest

 The authors declare no conflict of interest.
